# Validation of established thyroid ultrasound volume norms in a Chernobyl cohort

**DOI:** 10.1530/ETJ-25-0085

**Published:** 2025-07-01

**Authors:** Lydia B Zablotska, Robert J McConnell, Aleksandr V Rozhko, Patrick O'Kane, Vasilina Yauseyenka, Mark P Little, Victor Minenko, Vladimir Drozdovitch, Tamara Moskvicheva, Maureen Hatch, Tamara Yeudachkova, Kiyohiko Mabuchi, Elizabeth K Cahoon

**Affiliations:** ^1^ Department of Epidemiology and Biostatistics, School of Medicine, University of California, San Francisco, San Francisco, California, USA; ^2^ The New York Thyroid Center, Columbia University, New York, New York, USA; ^3^ Republican Research Center for Radiation Medicine and Human Ecology, Gomel, Belarus; ^4^ Department of Radiology, Thomas Jefferson University Hospital, Philadelphia, Pennsylvania, USA; ^5^ Radiation Epidemiology Branch, Division of Cancer Epidemiology and Genetics, National Cancer Institute, Bethesda, Maryland, USA; ^6^ Institute for Nuclear Problems, Belarusian State University, Minsk, Belarus

**Keywords:** Chernobyl nuclear accident, diffuse goiter, iodine deficiency, iodine radioisotopes, radiation, thyroid ultrasound volume

## Abstract

**Objective:**

To establish thyroid ultrasound volume norms appropriate for studies of diffuse goiter in a cohort of children and adolescents from an iodine-deficient population exposed to ^131^I by the Chernobyl fallout.

**Methods:**

A cohort of 11,970 Belarusians aged ≤18 years at the time of the 1986 Chernobyl accident with individual thyroid radiation dose estimates was screened 10–18 years later. From these, a low-dose subset of 2,392 with no thyroid diseases was selected to construct age- and sex-specific normative values for thyroid ultrasound volume, compared to Belarusian Ministry of Health (MOH) norms and existing WHO and European standards.

**Results:**

Cohort-specific values were generally lower than MOH norms and WHO standards for 11–17-year-olds. For those aged ≥18 years, internal norms were 30% higher in males and 15–30% lower in females than MOH norms, and exceeded European values for both sexes. Thyroid volume norms were about 40% higher in males and 30% higher in females as a function of BSA compared to European values. Thyroid volume continued to increase in both sexes, and by age 30–34 years, cohort-specific norms were 6% higher in males and 26% higher in females than European values. Urinary iodine concentration did not significantly explain variance in thyroid volume beyond sex, age, and BSA.

**Conclusions:**

In this iodine-deficient cohort of young Belarusians exposed to ^131^I by Chernobyl fallout, thyroid ultrasound volumes differed substantially from MOH norms and established WHO standards, prompting a revision of diffuse goiter definition using cohort-specific normative values.

## Introduction

The 1986 Chernobyl nuclear power plant accident exposed the residents of affected Belarusian territories to large amounts of radioiodines, primarily ^131^I, and was closely followed by an increase in the number of childhood thyroid cancers (1). A decade after the accident, the U.S. National Cancer Institute joined with the Health Ministries in Belarus and Ukraine to establish two parallel cohort studies, one in Belarus (Belarusian-American Cohort Study; BelAm) and the other in Ukraine (Ukrainian-American Cohort Study; UkrAm), to systematically screen for thyroid disorders that developed among those exposed as children or adolescents. The BelAm cohort consisted of those who were ≤18 years of age at the time of the accident and who had direct thyroid radioactivity measurements taken within the next 2 months (2). Subsequent analyses documented a significant dose-dependent increased risk of thyroid cancer, follicular adenoma, and benign nodules (3, 4, 5), and a moderately increased thyroid volume in cohort members exposed to low levels of radiation (6).

Other studies examined the prevalence of iodine deficiency and functional thyroid disorders in the BelAm cohort in the years after the Chernobyl accident (7, 8). However, examining the relationship between radiation dose and diffuse goiter proved to be problematic, largely because the BelAm study protocol specified a hybrid definition, characterizing diffuse goiter according to both palpation grade and ultrasound volume compared to a reference range provided by the Belarusian Ministry of Health (MOH). Yet, even at the time of cohort screening, it was uncertain if the MOH norms were appropriate standards since it was not known how they had been developed or even if they applied to the cohort. While palpation is usually sufficient to determine thyroid enlargement in regions of severe iodine deficiency, measurement of thyroid volume by ultrasound is preferred in areas of mild-to-moderate deficiency, such as Belarus at the time of screening (7, 9, 10, 11). However, inter-observer variation in sonographic thyroid volume can be as much as 26% and it is widely recognized that genetic, nutritional, and developmental features in different populations can impact measurements (12, 13). Finally, since information concerning thyroid enlargement in irradiated populations has been sparse and inconsistent (14, 15, 16), it is unclear if analyses of diffuse goiter in the BelAm cohort should be based upon WHO normative values or internally established reference ranges (17, 18).

Because of uncertainty about how to define diffuse goiter and what normal reference range was appropriate for our cohort, we established internal normative values for ultrasound thyroid volume and compared them to both the Belarusian MOH norms and WHO and European standards that existed at the time of cohort screening. Although similar validation studies have been carried out in unexposed populations, we believe that this is the first time that normative thyroid volume norms have been defined for an iodine-deficient population exposed to environmental radiation.

## Materials and methods

### Cohort description and screening procedures

The design and methods of the BelAm study have been described in detail (2, 3). We identified 38,543 potential cohort members who were ≤18 years of age at the time of the Chernobyl accident and who had direct thyroid radioactivity measurements taken within the next 2 months. Beginning in 1996, we began to trace exposed cohort members and invite them for thyroid screening, and 11,970 were screened at least once from 1996 to 2004, with most examined between 1997 and 2001 (3). Local institutional review boards in Belarus (Institutional Review Board of Republican Research Center for Radiation Medicine and Human Ecology, Gomel) and the United States (Special Studies Institutional Review Board of the National Cancer Institute) approved the study protocol, and signed informed consent was obtained from study cohort members or their legal guardians for minors.

Although the study protocol called for biennial screening, the Belarusian MOH mandated that all cohort members under 18 years of age were to be seen annually (2). At the time of screening, study cohort members resided primarily in Minsk and Gomel oblasts (administrative areas similar in size to a state or province) and were examined either at medical centers in Minsk and Gomel cities or at local medical clinics visited by mobile teams (3). All examiners were blinded to radiation dose and screening was carried out according to a well-defined protocol that specified a thyroid ultrasonogram and palpation examinations carried out independently by an endocrinologist and an ultrasonographer (2).

Goiter grade on palpation was defined according to the WHO criteria: grade 0, no palpable enlargement; grade 1, enlarged thyroid, not visible when the neck is in the neutral position; and grade 2, enlarged thyroid, visible when the neck is in the neutral position (19). Thyroid volume was calculated using the equation of Brunn (20), where the volume of each lobe (mL) = anteroposterior diameter (depth, cm) × mediolateral diameter (width, cm) × craniocaudal diameter (length, cm) × 0.479, and the lobe volumes were summed. The isthmic volume was included if its thickness exceeded 5 mm and the percent deviation of total thyroid volume from a reference range provided by the Belarusian MOH was noted (2). Body surface area (BSA) was calculated as weight (kg) ^0.425^ × height (cm) ^0.725^ × 71.84 × 10^−4^ (10). Although only 0.7% of patients had weight and height information collected during the baseline screening when thyroid volume was measured, we were able to retrieve these data from the second screening cycle for 92% of subjects. The weight and height data were obtained about 2 years (median = 2.2 years, mean = 2.6 years) after the initial thyroid volume measurement at baseline screening. According to the protocol, the ultrasonographer did not disclose the results of the ultrasound or their palpation examination until the endocrinologist had completed examining the patient. The endocrinologist then compared the individual palpation results and attempted to resolve differences through discussion or joint re-examination of the subject to reach consensus. Unresolved discrepancies were handled by registering goiter grade 0 or grade 1 to the next higher level. The study endocrinologist based a final diagnosis of diffuse goiter on both palpation and ultrasound volume exceeding the Belarusian MOH norms and recorded it on a Final Endocrine Summary form. In addition, there could be no structural abnormalities in the thyroid gland and the patient should be euthyroid or hypothyroid with normal thyroid antibody levels.

### Laboratory investigations

Serum concentrations of thyroid-stimulating hormone (TSH), thyroglobulin (Tg), free thyroxine, and antibodies to thyroid peroxidase were measured according to the manufacturers’ instructions. Urinary iodine concentrations were measured from spot urine samples photometrically using the Sandell–Kolthoff reaction as modified by Dunn (21).

### Dosimetry

The methods used to estimate individual radiation exposure have been described in detail (22). About 92% of the total thyroid dose received after the Chernobyl accident resulted from ^131^I that was ingested largely in tainted cow’s milk (23). Individual doses and their uncertainties were based on: i) direct thyroid measurements taken within 2 months after the accident; ii) ecological and biokinetic models that were used to assess the temporal variation of ^131^I activity in the thyroid; and iii) personal interview information on individual residential history, dietary habits, and administration of stable iodine to block the uptake of ^131^I by the thyroid. The dose reconstruction model accounted for age-specific thyroid volumes (24) based upon the measurements performed in 1991–1996 by the Sasakawa Memorial Health Foundation (25) and for the contribution of external and internal radioactive contamination of the body and clothes on the results of the direct thyroid measurements (24, 26, 27). The arithmetic mean of 1,000 individual stochastic doses due to ^131^I exposure calculated for each cohort member was used in all statistical analyses (22).

### Statistical analysis

Normal distribution of thyroid volume was assessed using the Kolmogorov–Smirnov test (*P* < 0.01). Since the distribution of thyroid volumes in the study population was right-skewed, this variable was log-transformed, which removed most of the skewness and kurtosis, leaving a nearly normal distribution for all ages and both sexes (*P* > 0.15). Log-transformed data were used to calculate percentiles based on the normal distribution, which were then transformed back to the linear scale. The medians and 95% confidence intervals of thyroid volume were calculated separately for sex, age and BSA categories and expressed as 10th, 25th, 50th, 75th and 97th percentile (97P) values. The Wilcoxon rank-sum and Kruskal–Wallis tests were used to compare the distribution of thyroid volume across categories of sex, age, BSA and urinary iodine concentration. The data for both sexes were separately fitted. Correlation between non-normally distributed continuous variables was assessed using the Spearman rank correlation test. The formula from Andersson *et al.* (28) was used to estimate the number of spot urine samples needed to assess population iodine levels with 95% confidence and a precision of ±15%. Analysis of covariance (ANCOVA, type III sum of squares) was employed to evaluate the extent to which categorical variables explain a significant proportion of the variance in thyroid volume. All analyses were performed using the SAS statistical software (29).

## Results

### Sample selected to establish internal thyroid volume norms

**Figure 1:**
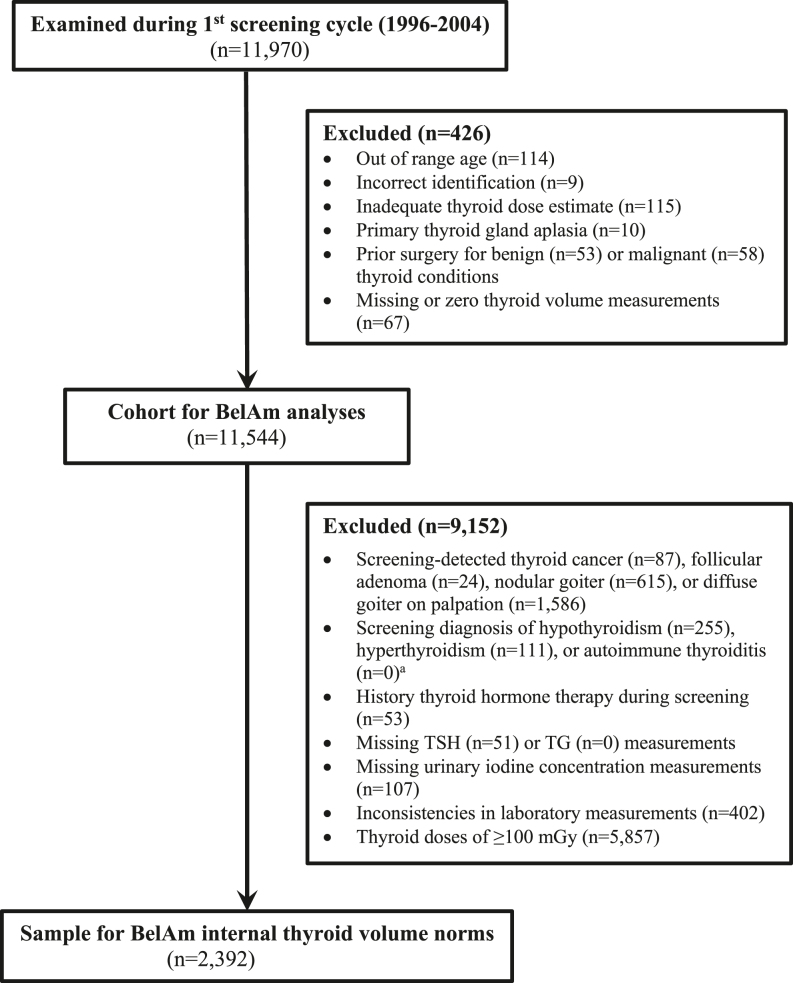
Flowchart for sample selection.

Out of 11,970 cohort members screened from 1996 to 2004, we excluded 426 due to incorrect identification or out-of-age range; inadequate thyroid dose estimate; primary thyroid gland aplasia; prior surgery for benign or malignant thyroid conditions; or missing or zero thyroid volume measurements, leaving a total of 11,544 cohort members. We further excluded 9,152 for screening-detected thyroid cancer, follicular adenoma, or nodular goiter; a diagnosis of diffuse goiter on palpation only; subclinical or overt hypo- or hyperthyroidism, or autoimmune thyroiditis; thyroid hormone therapy during screening; missing TSH or Tg measurements; missing urinary iodine concentration; inconsistencies in laboratory measurements; or thyroid doses ≥100 mGy. This left a reference population of 2,392 that was used to calculate the internal thyroid volume norms (Fig. 1).

Table 1 shows the estimated internal thyroid volume norms for those <18 years at screening in the BelAm cohort (*n* = 512, 21.4%). There were notable differences when these cohort-specific thyroid volume norms were compared to existing WHO and European standards and to the Belarusian MOH reference range used during screening. Up to age 13 years, the Belarusian MOH norms were identical to existing WHO standards for both males and females, before becoming uniformly larger, whereas the cohort-specific thyroid volume norms were generally lower than both the Belarusian MOH and WHO values throughout the entire age range for both sexes, particularly for prepubertal individuals (Table 1).

**Table 1:** Comparison of cohort-specific BelAm thyroid volume norms for those less than 18 years of age at screening with Belarusian Ministry of Health norms and normative values from an iodine-sufficient European population.

Population	97th percentile of thyroid volume (mL)							
	Male				Female			
	WHO/ICCIDD, 1997* (9)	Belarusian MOH, 1996	BelAm, 1996–2004	Zimmermann *et al.* 2004 (13)	WHO/ICCID, 1997* (9)	Belarusian MOH, 1996	BelAm, 1996–2004	Zimmermann *et al.* 2004 (13)
Sample size	1,793	Unknown	268	1,807	1,681	Unknown	244	1,807
Age, years								
11	9.0	9.0	4.51	5.34	10.4	10.4	7.99	5.73
12	10.4	10.4	8.76	6.03	11.7	11.7	8.75	6.59
13	12.0	14.0	13.61	N/A	13.1	13.8	13.20	N/A
14	13.9	16.2	14.34	N/A	14.6	16.7	14.75	N/A
15	16.0		15.02	N/A	16.1		16.8	N/A
16	N/A	20.7	18.64	N/A	N/A	17.3	17.18	N/A
17	N/A		20.58	N/A	N/A		18.04	N/A
Urinary iodine, μg/L^†^	>100	Unknown	73 (6–660)	203 (118–288)	(>100)	Unknown	85 (1–466)	203 (118–288)

BelAm, Belarusian-American Cohort Study of Thyroid Cancer and Other Thyroid Diseases; ICCIDD, International Council for Control of Iodine Deficiency Disorders; μg/L, microgram per liter; mL, milliliter; MOH, Ministry of Health; N/A, not available; WHO, World Health Organization.

* Includes children aged 6–15 years in 12 European countries.

^†^ Values are median (range).

In contrast, the internal norms for those aged 18 years or older (*n* = 1,880, 78.6%) were about 30% higher in males and 15–30% lower in females than the Belarusian MOH norms, but both exceeded contemporary European normative values (Table 2). It is notable that Belarusian MOH norms for women of this age group were higher than those for men, contrary to both our internal norms and the European standards. Thyroid volume continued to increase in both males and females up to and including the oldest age category. By ages 30–34 years, the cohort-specific norms were 6% higher for males and 26% higher for females than the European standards. The distribution of thyroid volume differed significantly by categories of sex and age (both *P* < 0.0001 from the Kruskal–Wallis Test, Table 2).

**Table 2:** Comparison of cohort-specific BelAm thyroid volume norms for those 18 years of age and older at screening with Belarusian Ministry of Health norms and normative values from an iodine-sufficient European population.

Population	97th percentile of thyroid volume (mL)					
	Male			Female		
	Belarusian MOH, 1996	European (30)	BelAm, 1996–2004	Belarusian MOH, 1996	European (30)	BelAm, 1996–2004
Sample size	Unknown	Unknown	888	Unknown	Unknown	992
Age, years		25			18	
18–19	17.3/18.4		20.21	20.7/24.0		17.20
20–24	18.4		23.78	24.0		18.85
25–29	20.3		26.06	25.3		19.76
30–34			26.44			22.69
*Urinary iodine, μg/L	Unknown	Unknown	76 (1–522)	Unknown	Unknown	74 (0.1–817)

BelAm, Belarusian-American Cohort Study of Thyroid Cancer and Other Thyroid Diseases; μg/L, microgram per liter; mL, milliliter; MOH, Ministry of Health.

* Values are median (range).

Table 3 presents the distribution of BSA and urinary iodine concentration by categories of attained age and sex for those aged older than 18 years. There was substantial correlation between thyroid volume and BSA (Spearman R correlation = 0.42, *P*-value<0.0001), but not between thyroid volume and urinary iodine (Spearman R correlation = −0.07, *P*-value = 0.0011). The average urinary iodine concentration at screening was below the sufficiency cutpoint of 100 μg/L for 60–70% of individuals across most age categories for both males and females. Females were more iodine-deficient at screning than males overall, but the difference was not statistically significant (P Wilcoxon rank-sum test = 0.56). The median urinary iodine concentrations were significantly different by categories of attained age among females only (P Kruskal–Wallis test = 0.0005), with the oldest (over 30 years) group having the lowest urinary iodine concentration value.

**Table 3:** Distribution of body surface area and urinary iodine concentration for those 18 years of age and older at screening by categories of attained age and sex, BelAm study sample.

Age, years	*n*	BSA, m^2^, mean (SD)	UI concentration, μg/L, median (IQR)	Urinary iodine distribution, %*				
				<20 μg/L	<50 μg/L	<100 μg/L	<300 μg/L	>300 μg/L
Males								
18–19	171	1.91 (0.16)	73.02 (42.61–129.75)	9%	34%	67%	97%	3%
20–24	318	1.92 (0.15)	79.79 (38.72–146.00)	12%	33%	60%	96%	4%
25–29	204	1.94 (0.15)	72.65 (37.70–142.90)	12%	36%	63%	96%	4%
30–34	77	1.91 (0.17)	59.43 (41.03–175.60)	12%	40%	61%	96%	4%
Females								
18–19	168	1.64 (0.13)	93.79 (52.31–166.30)	4%	26%	52%	92%	8%
20–24	362	1.66 (0.15)	75.39 (40.83–146.70)	8%	32%	62%	94%	6%
25–29	307	1.69 (0.15)	69.69 (43.25–129.20)	7%	31%	67%	97%	3%
30–34	92	1.69 (0.15)	53.80 (32.63–124.10)	13%	47%	68%	99%	1%

* Analysis of urinary iodine concentration was limited to 2,161 (90.3 %) observations due to 231 missing BSA values, as the data were categorized by BSA.

BelAm, Belarusian-American Cohort Study of Thyroid Cancer and Other Thyroid Diseases; BSA, body surface area; IQR, interquartile range (25–75%); SD, standard deviation.

Table 4 shows the estimated internal thyroid volume norms for categories of BSA, separately for males and females, among those aged older than 18 years and with urinary iodine >100 μg/L to avoid mixing iodine-sufficient and iodine-deficient samples. For similar BSA, females had consistently lower thyroid volume norms in the sample compared to males.

**Table 4:** 97th percentile thyroid volume by ultrasound, stratified by sex and BSA (m^2^) for those 18 years of age and older at screening with urinary iodine concentration >100 μg/L, BelAm study sample.

Population categories	Thyroid volume, mL					
	Guo *et al.* 2021 (29)			BelAm*		
	BSA, m^2^	P50	P97	BSA, m^2^	P50	P97
Total						
	<1.64	6.94	14.62	<1.62	10.83	19.71
	≤1.64 to <1.79	8.32	16.80	≤1.62 to <1.77	11.18	18.85
	≤1.79 to <1.94	9.79	19.77	≤1.77 to <1.91	12.66	19.93
	≥1.94	11.12	19.43	≥1.91	14.73	24.00
Males						
	<1.79	8.63	17.52	<1.81	12.63	22.05
	≤1.79 to <1.90	9.81	19.55	≤1.81 to <1.90	13.45	18.86
	≤1.90 to <2.02	10.62	20.18	≤1.90 to <2.01	14.49	24.08
	≥2.02	11.75	19.78	≥2.01	15.14	24.00
Females						
	<1.55	6.33	14.78	<1.56	10.1	17.26
	≤1.55 to <1.64	7.11	14.51	≤1.56 to <1.64	10.80	18.33
	≤1.64 to <1.73	7.74	15.27	≤1.64 to <1.76	11.23	18.94
	≥1.73	8.58	15.31	≥1.76	11.85	21.66

* Analysis was limited to 918 out of 2,392 (38.4 %) BelAm observations after excluding those aged 18 years and younger, those with urinary iodine concentrations ≤100 μg/L and those with missing BSA or urinary iodine concentration values.

BelAm, Belarusian-American Cohort Study of Thyroid Cancer and Other Thyroid Diseases; BSA, body surface area; *n*, sample size; P50, 50th percentile (median); P97, 97th percentile.

**Figure 2:**
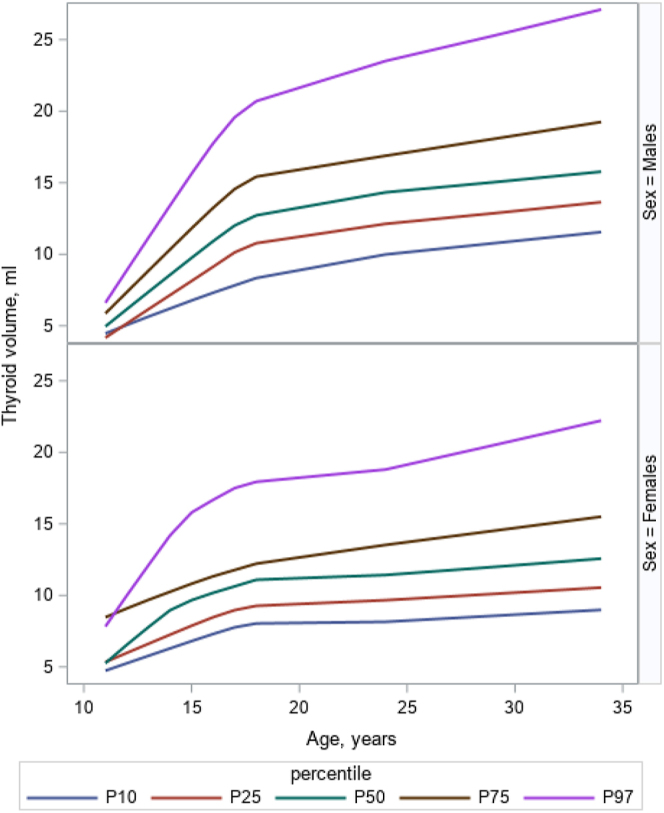
Total thyroid volume reference curves with common percentiles (P) for thyroid volume (mL) according to age (years), separately for males and females.

**Figure 3:**
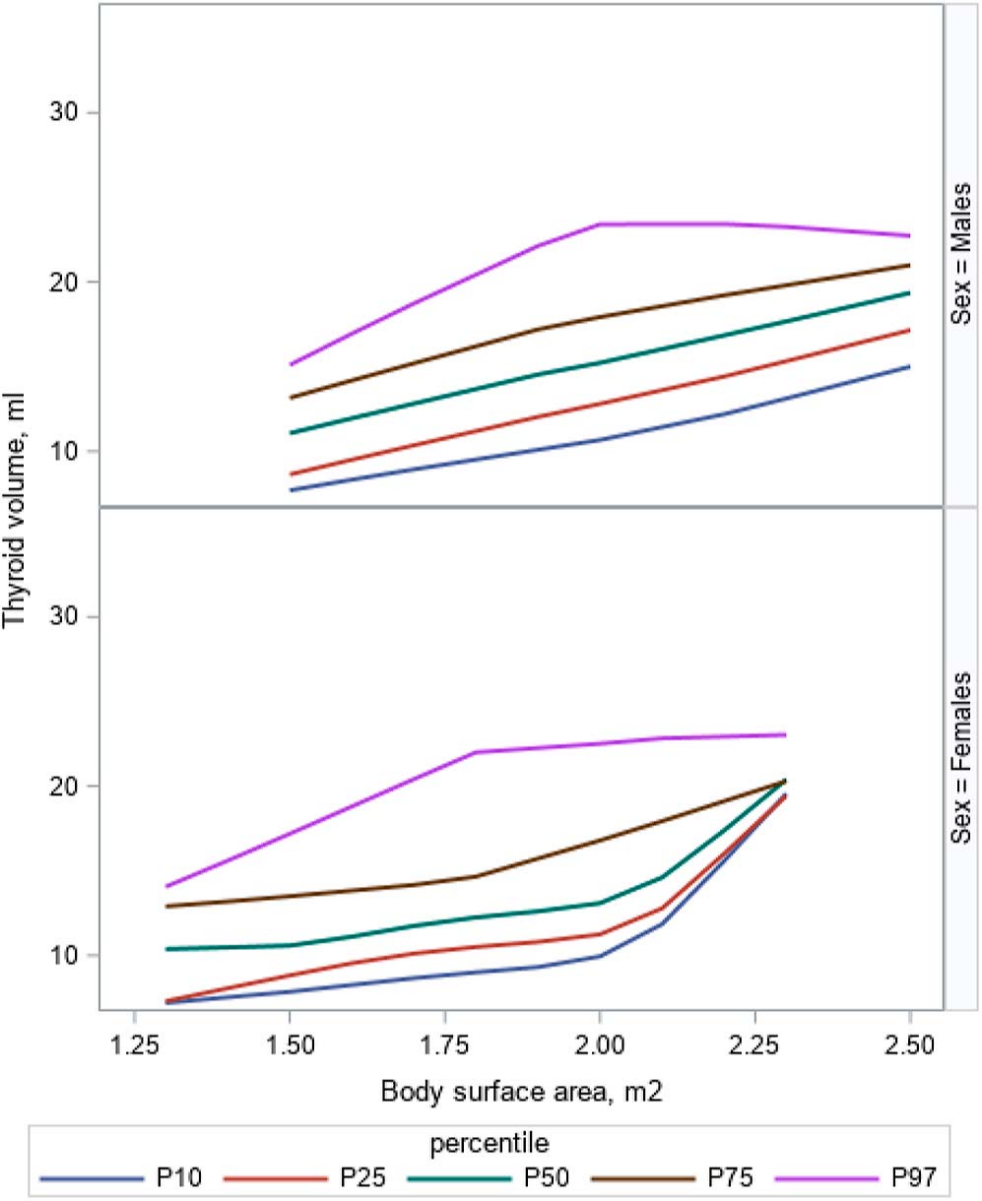
Total thyroid volume reference curves by body surface area (m^2^) with common percentiles (P), stratified by sex, for adults (>18 years).

Figure 2 illustrates an increase in thyroid volume norms with age and sex. Thyroid volume norms exhibited an exponential increase up to approximately 18 years of age, after which the rate of increase slowed significantly but continued to rise gradually until around 34 years of age. Figure 3 shows an increase in the thyroid volume with increasing BSA for adults (>18 years), separately for males and females, up until BSA of 2.0 and then a mostly stable thyroid volume.

Using analysis of covariance (type III sum of squares), we observed significant effects of sex, age and BSA on thyroid volume among adults (all *P* < 0.0001). Urinary iodine concentration did not account for a significant proportion of variance in thyroid volume beyond sex, age, and BSA (*P* = 0.14).

## Discussion

We believe that this is the first time that normative thyroid volume norms have been defined for an iodine-deficient population exposed to environmental radiation. Although WHO reference values have been traditionally used to define normal thyroid volume, these values may not be universally applicable due to genetic, nutritional, and developmental differences between populations (17, 18). It is also unclear what reference range is appropriate for an iodine-deficient irradiated group. Recognizing these uncertainties, we carried out a validation study using a low-dose subset of our cohort and compared the results to WHO and European standards and the Belarusian MOH norms.

For prepubertal children, our internal norms were smaller than those provided by the Belarusian MOH, which themselves mirrored WHO reference standards that represented ultrasound techniques and general European nutrition at the time of cohort screening (9). However, for those aged 13–17 years, our norms were more closely aligned with the Belarusian MOH guidelines, and both exceeded the WHO normative values. The internal norms for those aged 18 years or older were about 30% higher in males and 15–30% lower in females compared to the Belarusian MOH norms.

Current clinical guidelines for ultrasound thyroid volume in adults are based on age- and sex-specific thyroid volumes in a large sample of iodine-sufficient (mean urinary iodine >100 μg/L) adults from Sweden and Germany and recommend a fixed thyroid volume norm of 18 mL for females and 25 mL for males 18 years of age or older (30, 31). Several clinical studies recommend even lower norms, 10–15 mL for adult females and 12–18 mL for males (32, 33). In contrast to these established clinical guidelines, in our selected sample of iodine-deficient subjects from Belarus who had no thyroid diseases and were exposed to low or no radiation after the Chernobyl accident, thyroid volume continued to increase over the entire age range, and by age 30–34 years was 6% higher for males and 26% higher for females than the Western norms (Table 2) (30). Similar age-related increases in thyroid volume after age 18 years have also been noted in healthy iodine-sufficient Turkish and Chinese adults (34, 35, 36). Thus, reliance on a single standard thyroid volume norm for those 18 years of age or older could lead to errors in diagnosis of diffuse goiter.

Thyroid volume depends on a number of anthropometric factors in addition to age, such as sex, weight, height, body mass index, body fat content, and BSA, which shows the strongest correlation (37, 38). In addition to using an internal reference range based upon attained age and sex, similar to other epidemiological studies of diffuse goiter (39, 40), we also estimated norms based on BSA. Previous studies have suggested that sex-specific thyroid volume differences could be explained by puberty-related physical changes due to an increase in BSA. Table 4 shows thyroid volume norms for adult study subjects with urinary iodine >100 μg/L to avoid mixing iodine-sufficient and iodine-deficient samples. The distribution of BSA in our study closely matched that of the large population-based sample from China, with nearly identical quartile cutpoints (35). For all quartiles of BSA, thyroid volumes were higher (up to twice as much) in our patients with ‘normal’ urinary iodine concentration. It is possible that these subjects were iodine deficient in the past and that their goiters did not fully regress after they became more iodine sufficient. Azizi *et al.* showed that the goiter rate in adults may remain elevated for a number of years following introduction of iodized salt (41). These findings further support our conclusion that locally established norms are preferable to international ones for populations with current or past iodine deficiency.

We found significantly different median urinary iodine concentrations by categories of attained age among females only (Table 3). According to the formula of Andersson *et al.* (28), a sample size of 131 would be necessary to assess the population iodine levels with 95% confidence and a precision of ±15%. All cell frequencies in Table 3 exceeded this threshold except for the 30–34-year age group. Thus, we had sufficient sample size to evaluate meaningful differences in urinary iodine concentration between the categories.

We have previously examined urinary iodine in this cohort and concluded that it varied by sex, oblast of residence and type of residence (lower in rural than in urban residents) (7). A nation-wide iodination program was announced in March 2000, but the use of iodized salt in the production of processed foods in Belarus was not introduced until April 2001. Most of our sample subjects were screened before April 2001 (3), which might explain low urinary iodine concentrations (medians for spot urine analyses for males and females of both age categories <100 μg/L).

In addition to being moderately iodine deficient, our cohort was irradiated, and the association between radiation exposure and thyroid enlargement has been systematically examined in only a few studies, and then with largely inconsistent findings (14, 15, 16). A study of atomic bomb survivors from Nagasaki reported no increase in the risk of diffuse goiter (14), while increased risk was reported after childhood exposure to Chernobyl fallout (15) and in those externally irradiated in childhood for the treatment of hemangioma (16). However, all these studies used different definitions and diagnostic methods and examined populations with widely different levels of iodine intake. Our recent analysis of the association between radiation dose and thyroid volume in the BelAm cohort found a modest positive association for doses below 0.15 Gy in a subgroup that was 18 years or older at the time of screening (6).

The major strengths of our study are the availability of individual ^131^I thyroid doses based on direct thyroid radioactivity measurements made within 2 months of the accident and the completion of all procedures according to a well-defined and standardized protocol that blinded all examiners to dose (2). To our knowledge, it is the largest study of subjects who were exposed to environmental doses of radioiodines as children or adolescents and were prospectively screened for thyroid diseases. Although weight and height measurements were collected several years after baseline thyroid volume was measured, the correlation between weight and height measurements taken at both baseline and second screening was very good (Pearson R correlation = 0.92) for a sample of 15 patients. At the time of the first screening, 1,880 out of 2,392 study subjects (78.6%) were adults (over 18 years old). Therefore, we considered height measurements from the second screening, conducted 2 to 3 years later, to be appropriate for this subgroup. While we acknowledge that weight may fluctuate in young adults, we believe these changes are unlikely to meaningfully affect BSA-based analyses. Supporting this assumption, Figure 5 in Redlarski *et al.* (42) shows that BSA estimates using the DuBois formula change minimally with a weight gain of 5–10 kg, a range that likely overestimates actual weight variation in our cohort over the 2- to 3-year interval. In addition, since palpation can misrepresent thyroid size in up to 24% of mildly iodine-deficient children (43), both under- and over-estimating thyroid ultrasound volume, the effects on the cohort-specific norms are uncertain.

In summary, normative ranges for thyroid volume vary across populations and over time, and there is a growing consensus that locally established norms are preferred. In addition, it is not known what reference values are suitable for an iodine-deficient irradiated group. To address these uncertainties, we constructed age- and sex-specific internal norms using a low-dose subset of the BelAm cohort. Because our cohort-specific values differed substantially from both WHO standards and the Belarusian MOH norms used during cohort screening, we revised our definition of diffuse goiter using the new internal volume measurements.

## Declaration of interest

The authors declare that there is no conflict of interest that could be perceived as prejudicing the impartiality of the work reported.

## Funding

Funding for this study was provided by the National Cancer Institutehttps://doi.org/10.13039/100000054 grant CA132918 to LBZ, contract NO1-CP-21178 to LBZ, RJM, and PO, and the Intramural Research Program of the Division of Cancer Epidemiology and Genetics of the National Cancer Institute. The U.S. Department of Energy provided funding at the earlier stages of the study and the Nuclear Regulatory Commission provided the initial funds for purchase of equipment, but neither of the two agencies had any role in the design and analysis of the study.

## Author contribution statement

LZ conceptualized the study and participated in data collection, methodology, statistical analysis and writing of the manuscript. RM helped in data collection and writing. AR helped in data collection and revision of the final version of the manuscript. PO helped in data collection and writing; VYa helped in data collection and revision of the final version of the manuscript. ML helped in writing, methodology and statistical analysis. VM helped in data collection and revision of the final version of the manuscript. VD helped in data collection, methodology and writing. TM helped in data collection and revision of the final version of the manuscript. MH helped in data collection and writing. TYe helped in data collection and revision of the final version of the manuscript. KM helped in data collection, methodology and writing. EC helped in methodology, writing, reviewing and editing.

## Data availability

The dataset analyzed during the current study is not publicly available due to individual participant confidentiality. All inquiries about the data should be directed to Dr E Cahoon.
